# Cluster headache and kynurenines

**DOI:** 10.1186/s10194-023-01570-9

**Published:** 2023-04-05

**Authors:** Bernadett Tuka, Tamás Körtési, Nikolett Nánási, Ferenc Tömösi, Tamás Janáky, Dániel Veréb, Délia Szok, János Tajti, László Vécsei

**Affiliations:** 1 ELKH-SZTE Neuroscience Research Group, Department of Neurology, Faculty of Medicine, University of Szeged, Semmelweis U 6, Szeged, Hungary 6725; 2 Department of Radiology, Faculty of Medicine, University of Szeged, Szeged, Hungary; 3 Faculty of Health Sciences and Social Studies, University of Szeged, Szeged, Hungary; 4 Department of Medical Chemistry, Interdisciplinary Excellence Centre, University of Szeged, Szeged, Hungary; 5 Department of Neurology, Albert Szent-Györgyi Medical School, University of Szeged, Szeged, Hungary

**Keywords:** Episodic cluster headache, Interbout and ictal periods, Plasma kynurenine metabolites, Clinical features

## Abstract

**Background:**

The glutamatergic neurotransmission has important role in the pathomechanism of primary headache disorders. The kynurenine metabolites derived from catabolism of tryptophan (Trp) have significant involvement not only in glutamatergic processes, but also in the neuroinflammation, the oxidative stress and the mitochondrial dysfunctions. Previously we identified a depressed peripheral Trp metabolism in interictal period of episodic migraineurs, which prompted us to examine this pathway in patients with episodic cluster headache (CH) as well. Our aims were to compare the concentrations of compounds both in headache-free and attack periods, and to find correlations between Trp metabolism and the clinical features of CH. Levels of 11 molecules were determined in peripheral blood plasma of healthy controls (*n* = 22) and interbout/ictal periods of CH patients (*n* = 24) by neurochemical measurements.

**Findings:**

Significantly decreased L-kynurenine (KYN, *p* < 0.01), while increased quinolinic acid (QUINA, *p* < 0.005) plasma concentrations were detected in the interbout period of CH patients compared to healthy subjects. The levels of KYN are further reduced during the ictal period compared to the controls (*p* < 0.006). There was a moderate, negative correlation between disease duration and interbout QUINA levels (*p* < 0.048, *R* =  − 0.459); and between the total number of CH attacks experienced during the lifetime of patients and the interbout KYN concentrations (*p* < 0.024, *R* =  − 0.516). Linear regression models revealed negative associations between age and levels of Trp, kynurenic acid, 3-hdyroxyanthranilic acid and QUINA in healthy control subjects, as well as between age and ictal level of anthranilic acid.

**Conclusions:**

Our results refer to a specifically altered Trp metabolism in CH patients. The onset of metabolic imbalance can be attributed to the interbout period, where the decreased KYN level is unable to perform its protective functions, while the concentration of QUINA, as a toxic compound, increases. These processes can trigger CH attacks, which may be associated with glutamate excess induced neurotoxicity, neuroinflammation and oxidative stress. Further studies are needed to elucidate the exact functions of these molecular alterations that can contribute to identify new, potential biomarkers in the therapy of CH.

**Supplementary Information:**

The online version contains supplementary material available at 10.1186/s10194-023-01570-9.

## Introduction

Cluster headache (CH) is a primary trigeminal autonomic cephalalgia characterised by extremely grievous, strictly unilateral pain localized around the orbital, supraorbital or temporal areas. The headache attack lasts for 15 to 180 min and is associated with, among other things, conjunctival injection, lacrimation, eyelid edema, nasal congestion or rhinorrhea [1–3]. Although the correct pathomechanism of CH is unclear, but anatomical connections between the hypothalamus, the trigeminovascular unit and the parasympathetic nervous system, as well as the molecular changes they define, are crucial in the development of headache disease [4, 5]. The circannual/circadian periodicity of CH and altered secretions of certain hormones (e.g. cortisol, melatonin) indicate the involvement of hypothalamus [6]*.* Activation of some elements of the trigeminovascular system (e.g. ophthalmic branch of the trigeminal ganglion) has been observed during CH, in which a number of neuropeptides—e.g. calcitonin gene-related peptide, vasoactive intestinal polypeptide and pituitary adenylate-cyclase activating polypeptide (PACAP)—involved and have a privileged role forming the parasympathetic cranial symptoms [7, 8]. In addition to neuropeptides, the glutamate is the other key molecule, which also receives special attention in the pathomechanism of primary headaches, especially in migraine [9]. The altered glutamate (Glu) neurotransmission, subsequently the excitotoxicity caused oxidative stress and hyperexcitabilty, may play role in the initiation of attacks***.*** Since Glu acting at N-methyl-D-aspartate (NMDA) receptors, it plays a key role in the induction of nociceptive sensitization [10]. This suggests, that alterations in NMDA receptor signaling or in the endogenous machinery that activates NMDA receptors may be relevant to the pathophysiology of CH. It is consistent with this hypothesis that memantine, a fast off-rate NMDA-gated ion channel blocker, has shown efficacy in reducing CH attacks in resistant patients, even if clinical studies are still limited [11]. Endogenous regulators of glutamatergic neurotransmission include certain metabolites of the kynurenine pathway (KP), which evolved from tryptophan (Trp) catabolism. Certain metabolites of pathway are neuroactive and play crucial roles in the modulation of NMDA receptor function. Since glutamate receptors induced overexcitation has essential role in the development of several neurological disorders, the KP has recently become the subject of intense investigations [12, 13]. However, mainly migraine studies and their results are available: we have no knowledge of animal studies with CH and there are limited clinical data are on kynurenines and CH. In our previous preliminary study altered kynurenine metabolism was detected in a special animal model of headache. In addition to Glu and serotonin, the orofacial Complete Freund’s Adjuvant induced elevated kynurenic acid (KYNA) and L-kynurenine (KYN) concentrations in the brainstem [14]. Electrical stimulation of the trigeminal ganglion caused overexpressed PACAP levels in the area of trigeminal nucleus caudalis in rat that KYNA was able to protect [13]. The role of kynurenines was examined in our episodic migraine study. In our previous examination, peripheral plasma samples of were remarkably decreased peripheral Trp pathway was identified in attack free period of episodic migraineurs compared to healthy control subjects. Especially, the levels of Trp, KYN, KYNA, anthranilic acid (ANA), picolinic acid (PICA) and 5-hydroxyindoleaceticacid (5-HIAA) showed significant reduction in the interictal phase. Moreover, some associations between metabolic alterations and clinical features of migraine were also detected in this study [15]. However, so far only one study examined the role of KP in CH patients. Curto and her co-workers found decreased KYN, KYNA, 3-hydroxykynurenine, 3-hydroxyanthranilic acid (3-HANA), xanthurenic acid (XA), 5-HIAA and quinolinic acid (QUINA) levels, but significantly increased Trp and ANA concentrations in the serum of the overall population of patients affected by CH (episodic and chronic). Difference between the chronic and episodic CH groups was only in the level of KYNA, which was higher in patients with chronic CH [16]. In light of these data, we were curious about how the proportion of major Trp metabolites in the blood of our CH patients changes depending on their headache period and other clinical features.

Our aims were:

- to determine the concentrations of 11 metabolites of Trp pathway in the peripheral plasma of episodic CH patients compared to healthy control subjects.
- to differentiate between metabolic alterations in the interbout/ictal periods of patients
- to describe the relationship between altered Trp metabolism and clinical parameters of the disease/attacks.

## Materials and methods

### Participants

All patients enrolled in this study are treated as outpatients at the Department of Neurology, Albert Szent-Györgyi Medical School, University of Szeged. Examinations were conducted after the approval of the local Ethical Committee of the University of Szeged (87/2009) and the Department of Health Administration of National Public Health Centre (29022–5/2019/EÜIG, 28324–5/2019/EÜIG) adhering to the most recent revision of the Declaration of Helsinki.

Inclusion criteria: Episodic cluster headache patients (CH, *n* = 24) fulfilling the criteria of the 3^rd^ edition of The International Classification of Headache Disorders were registered, and healthy control subjects (*n* = 22) were recruited. In order to keep the groups as homogenous as possible, they were matched in terms of age and sex (25–55 years, female and male in a similar proportion in both groups).

Peripheral blood samples were collected from the cubital vein of patients during the interbout (attack free) and ictal (attack) periods, and from healthy controls on one occasion. Interbout phase was defined as headache-free period at least 1 week after the last attack. EDTA containing blood collection tubes (BD Vacutainer K2E 6 ml) were used to obtain the samples between 8:30 a.m. and 3:30 p.m. Plasma samples were separated (3000 rpm at 4 °C for 15 min) and stored at − 80 °C until determination of Trp metabolites by ultrahigh-performance liquid chromatography–tandem mass spectrometry (UHPLC–MS/MS). Samples were coded to allow for blind measurements.

From among the 24 CH patients 19 samples were acquired during the interbout phase and 11 samples were acquired during the ictal phase. Self-controlled paired samples were taken from 6 CH patients during both periods, so 13 interbout samples derived from different patients (3 groups comparison). During headache attacks, patients were asked not to take their usual painkillers or specific attack medication until blood samples were taken. There were no restrictions regarding food and drink intake. Exclusion criteria for both the CH and control group included the presence of other type of headache (e.g. tension type headache) less than 48 h before sampling or other chronic pain conditions related to traumatic events or serious systemic disorders such as cardiovascular and metabolic diseases, immunological, neurological disorders, as well as clinically diagnosed psychiatric disorders. Subsequently, using any kind of chronic medication was also not allowed. Detailed questionnaires were taken from CH patients regarding the duration of disease, the frequency of attacks, the duration of clusters, the previous cluster period before sampling, the onset of cluster during ictal cupping and the onset of attack. Table 1 contains relevant demographic and clinical data.

**Table 1:** General and clinical data of cluster headache patients and healthy control subjects (mean ± standard deviation)

	**Episodic cluster headache patients**	**Healthy control subjects**
**Gender**	Male (*n* = 21)	Male (*n* = 18)
	Female (*n* = 3)	Female (*n* = 4)
**Age (years)**	36.58 ± 8.62	32.75 ± 10.79
**Number of subjects in different groups and number of samples in different periods of headache**	**Patients, *n* = 24** Interbout sample, *n* = 19 Ictal sample, *n* = 11 Paired samples, *n* = 6	**Subjects, *n* = 22** No regular headache syndromes, chronic diseases and drugs
**Clinical data of Cluster headache patients (interbout and ictal samples)**		
**Disease Duration (years)**	5.79 ± 4.53 and 11.18 ± 6.98	
**Attack frequency (attack/year)**	1.32 ± 0.58 and 1.45 ± 0.52	
**Duration of cluster period (days)**	35.95 ± 43.84 and 49.36 ± 47.20	
**Last attack before the interbout cupping (days)**	152.05 ± 197.70	
**Beginning of attack until ictal cupping (hours)**	4.18 ± 6.64	
**Start of cluster period during ictal sampling (days)**	21.64 ± 17. 06	
**Previous last attack before the ictal sampling (days)**	198.50 ± 244.47	

### Quantitative determination of Trp metabolites by UHPLC–MS/MS method

For the chemical analysis of KP a previously developed UPLC–MS/MS method was utilized [15] with some modification. This technique was successfully adapted to an ACQUITY H-Class UPLC™ liquid chromatography system equipped with Xevo TQ-S micro Triple Quadrupole Mass Spectrometer (Waters, Manchester, UK) applying the appropriate preparations and settings. Three of the 11 metabolites of interest (QUINA, PICA, and XA) were quantified in form of their butylated derivatives. Although no validation process was carried out on this device, quality control (QC) samples were prepared and measured during the analysis and some samples were tested in the original setup as well. As a result of the latter one, the obtained values were completely matched with each other, and furthermore 3-HK metabolite can be quantified in a lower concentration range with Xevo TQ-S. All samples were measured in duplicates, except QC samples (15–15 replicates for control and cluster headache groups).

### Statistical analysis

We performed statistical analysis forming 2 groups: healthy control (*n* = 22) and interbout CH patients (*n* = 19), then forming 3 groups: healthy control (*n* = 22), interbout CH patients (*n* = 13 independent samples) and ictal CH patients (*n* = 11). In all cases, the normality and homogeneity of variance were tested; afterwards, Mann–Whitney U and Kruskal–Wallis tests were run. The effect of clinical parameters (age, disease duration, attack frequency, cluster duration, onset of cluster and attack, previous clusters, etc.) on the metabolic changes was investigated in the interbout and ictal subgroups using linear regression models. Mean and standard deviation values ​​were indicated in the description of the results, while median of data and value of interquartile range (IQR) were represented in the figures. Significance level was accepted at *p* < 0.05.

To confirm the results of the univariate statistical analyses, we performed an additional multivariate analysis to assess whether the altered metabolite profile of the tryptophan pathway observed in cluster headache patients is able to accurately distinguish them from the healthy control group. To do this, we chose a partial least squares (PLS) model extended with a linear discriminant analysis (LDA) using the latent variables acquired from the PLS analyses [17], a model that performed well in our previous study [15]. In addition to metabolite levels, age was included as a confound variable in the model to assess how it affects metabolite levels. Classification was performed between cluster interbout vs. healthy, cluster ictal vs. healthy and cluster ictal vs. cluster interbout groups. The PLS-LDA analysis was performed in Matlab R2021 (MathWorks, inc.) using the libPLS package.

## Results

### Differences in plasma levels of Trp metabolites between interbout/ictal periods of CH patients and healthy controls

**Fig. 1:**
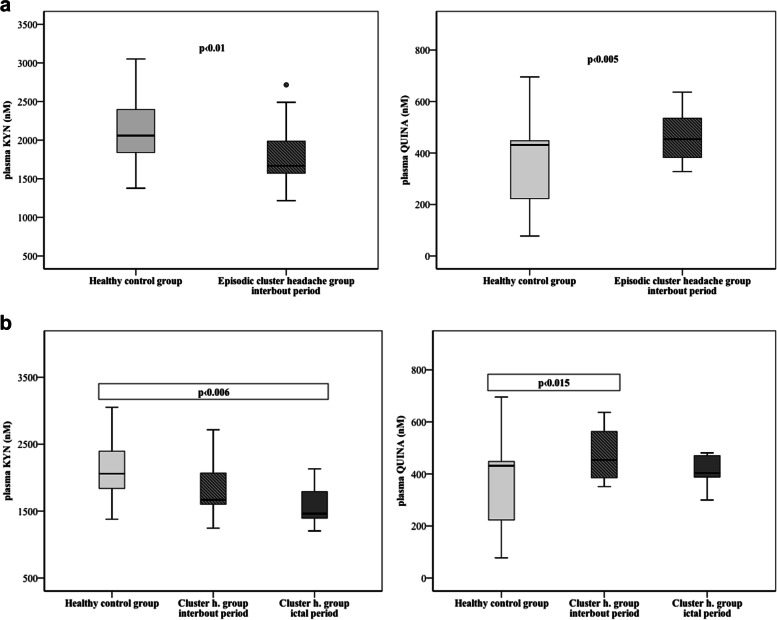
**a** and **b** Significant changes of KYN and QUINA plasma levels in the interbout/ictal periods of CH patients compared to healthy control subjects. Median of data and value of interquartile range were represented in the figures. KYN: L-kynurenine, QUINA: quinolinic acid

Compared to the healthy control group (*n* = 22) we detected significantly lower plasma concentrations of KYN (2150.22 ± 474.83 vs. 1771.38 ± 399.71; *p* < 0.01), but higher QUINA levels (361.08 ± 164.89 vs. 463.39 ± 96.36; *p* < 0.005) in the interbout phase of CH patients (*n* = 19) (Fig. 1a). In the analysis of 3 groups (control, *n* = 22; interbout, *n* = 13; ictal, *n* = 11), the level of KYN decreased further in the ictal period (1581.99 ± 328.13; *p* < 0.006). The significant QUINA increase was maintained in the attack-free period compared to controls (475.91 ± 103.72; *p* < 0.015), however its concentrations returned below the control value during attacks (Fig. 1b, Table 2). The interbout data of 6 CH patients, whose plasma samples were collected from both periods, were excluded from this statistical analysis in order to create independent groups. Other metabolites did not show any significant differences between groups. Details are included in Table 3.

**Table 2:** Plasma concentrations of Trp metabolites in healthy controls and interbout period of cluster headache patients

**Metab. (nM)**	**Groups: median** ± **IQR**		***p*-values between groups**
	**1. Healthy control (*n* = 22)**	**2. Cluster interbout (*n* = 19)**	
**Trp**	57,737.78 ± 21,948.78	50,965.09 ± 18,854.21	0.601
**KYN**	2059.42 ± 720.39	1668.05 ± 526.45	**0.010**
**KYNA**	43.56 ± 26.30	39.49 ± 16.14	0.497
**ANA**	44.18 ± 34.48	35.25 ± 20.56	0.229
**3-HK**	41.03 ± 15.78	44.31 ± 12.96	0.530
**XA**	15.41 ± 10.81	13.90 ± 8.92	0.174
**3-HANA**	49.18 ± 32.17	53.69 ± 31.54	0.320
**PICA**	48.20 ± 34.74	34.96 ± 24.57	0.296
**QUINA**	431.58 ± 233.42	453.82 ± 180.95	**0.005**
**5-HT**	43.72 ± 311.28	216.34 ± 229.24	0.157
**5-HIAA**	42.59 ± 21.55	43.89 ± 191.80	0.143

Significant differences were revealed between control and interbout data in cases of KYN and QUINA

*IQR* interquartile range, *Metab* metabolites, *Trp* Tryptophan, *KYN* L-kynurenine, *KYNA* Kynurenic acid, *ANA* Anthranilic acid, *3-HK* 3-hydroxikynurenine, *XA* Xanthurenic acid, *3-HANA* 3-hydroxianthranilic acid, *PICA* Picolinic acid, *QUINA* Quinolinic acid, *5-HT* 5-hydroxytryptamine, *5-HIAA* 5-hydroxiindoleaceticacid

**Table 3:** Plasma concentrations of Trp metabolites in healthy controls and cluster headache patients in both interbout and ictal periods

**Metab. (nM)**	**Groups: Median** ± **IQR**			***p*-values between groups**
	**1. Healthy Control (*n* = 22)**	**2. Cluster Interbout (*n* = 13)**	**3. Cluster Ictal (*n* = 11)**	
**Trp**	57,737.78 ± 21,948.78	57,696.19 ± 20,535.86	49,724.64 ± 16,000.00	0.839
**KYN**	2059.42 ± 720.39	1668.05 ± 570.59	1462.54 ± 578.66	**1–3: 0.006**
**KYNA**	43.56 ± 26.30	41.56 ± 25.90	32.60 ± 14.11	0.183
**ANA**	44.18 ± 34.48	35.25 ± 23.61	28.12 ± 19.57	0.164
**3-HK**	41.03 ± 15.78	44.31 ± 16.11	33.92 ± 35.60	0.643
**XA**	15.41 ± 10.81	16.00 ± 10.86	11.80 ± 8.97	0.247
**3-HANA**	49.18 ± 32.17	53.61 ± 32.98	45.60 ± 23.86	0.779
**PICA**	48.20 ± 34.74	39.01 ± 27.71	31.43 ± 16.69	0.259
**QUINA**	431.58 ± 233.42	453.82 ± 206.39	403.35 ± 109.50	**1–2: 0.015**
**5-HT**	43.72 ± 311.28	221.98 ± 360.68	366.25 ± 412.22	0.070
**5-HIAA**	42.59 ± 21.55	46.71 ± 10.66	43.21 ± 12.89	0.124

All p-values were added in the table between control and headache periods of cluster patients. Significant differences were revealed between control and ictal data in case of KYN and between control and interbout data in case of QUINA

*IQR* interquartile range, *Metab* metabolites, *Trp* Tryptophan, *KYN* L-kynurenine, *KYNA* Kynurenic acid, *ANA* Anthranilic acid, *3-HK* 3-hydroxikynurenine, *XA* Xanthurenic acid, *3-HANA* 3-hydroxianthranilic acid, *PICA* Picolinic acid, *QUINA* Quinolinic acid, *5-HT* 5-hydroxytryptamine, *5-HIAA* 5-hydroxiindoleaceticacid

**Fig. 2:**
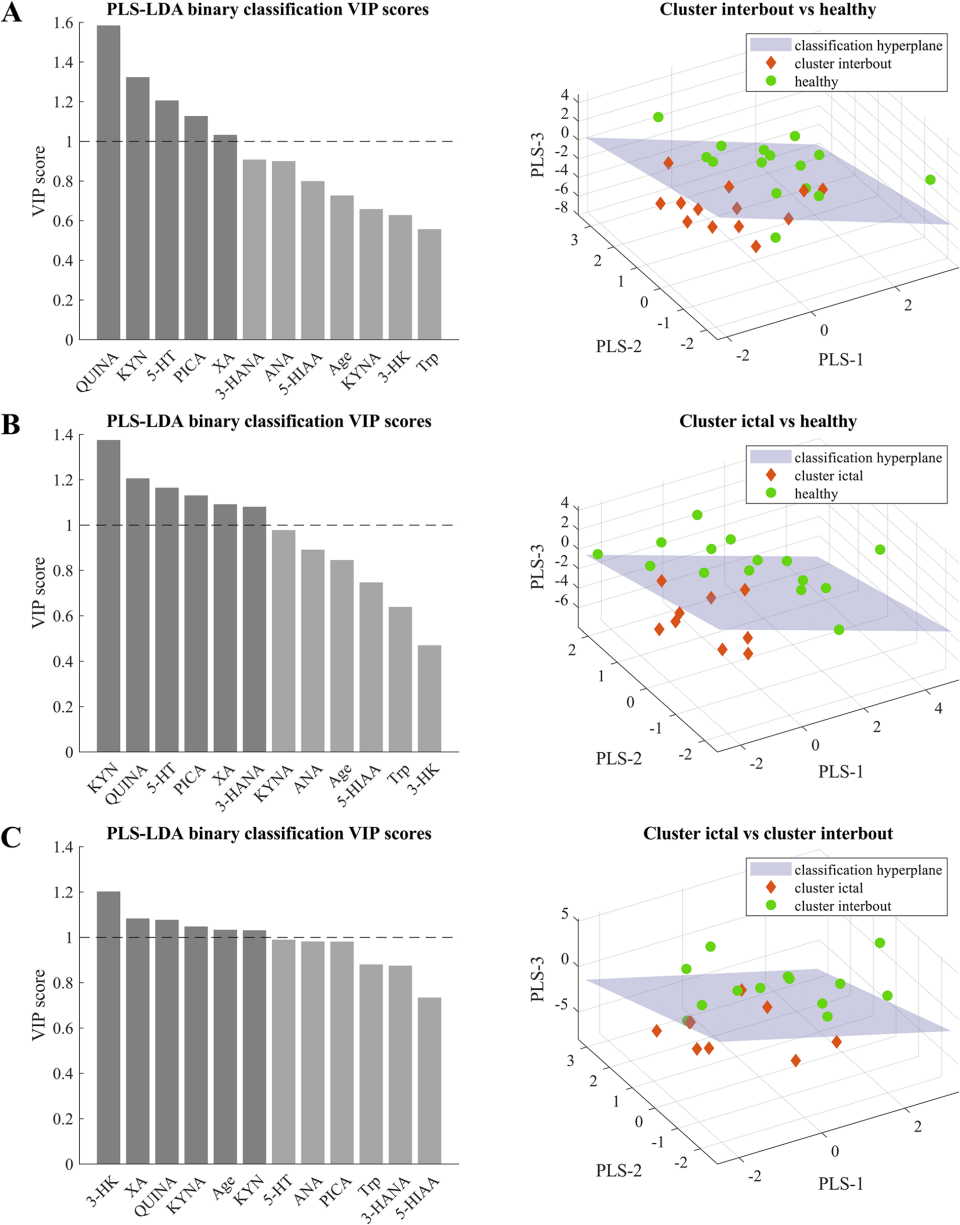
**a**, **b** and **c** Linear relationships between the disease duration and plasma levels of interbout QUINA, as well as the total number of CH attacks and plasma levels of interbout KYN. Median of data and value of interquartile range were represented in the figures. KYN: L-kynurenine, QUINA: quinolinic acid

The PLS-LDA model achieved the best classification results with 3 latent variables extracted in the PLS decomposition, with a sensitivity of 69.23%, a specificity of 93.33% and an AUC of 0.93 (RMSE = 0.18) in the comparison of cluster interbout (*n* = 13) vs. healthy control (*n* = 15) (Fig. 2a); with a sensitivity of 77.78%, a specificity of 100% and an AUC of 0.98 (RMSE = 0.14) in the comparison of cluster ictal (*n* = 9) vs. healthy control (*n* = 15) (Fig. 2b); with a sensitivity of 77.78%, a specificity of 92.31% and an AUC of 0.94 (RMSE = 0.14) in the comparison of cluster ictal (*n* = 9) vs. cluster interbout (*n* = 13) (Fig. 2c). Metabolites which proved most definitive in the classification (chosen as having a VIP of > 1; see the Supplementary files 1 and 2 for further details) were similar to those that showed alterations in the CH group, namely KYN and QUINA. In addition, the 5-hydroxytryptamine (5-HT), PICA, XA and 3-HANA seem to be remarkable predictors in comparisons of interbout/ictal vs. healthy control, while in CH patients the metabolic changes between the interbout and headache phases may be defined by the 3-HK, XA, QUINA, KYNA, KYN and the age.

Because of the relative scarcity of cluster headache patients in general, we conducted a post-hoc power analysis for the multivariate analysis to assess its vailidity using the area under the curve (AUC) as effect size for the classification. Using the method described in [18], we found that, compared to a null hypothesis of AUC = 0.5, the statistical power for detecting an AUC of 0.9 in all three classification analyses would be over 99%.

### Relationship between altered plasma Trp metabolites in the interbout/ictal periods of patients and clinical features of CH

**Fig. 3:**
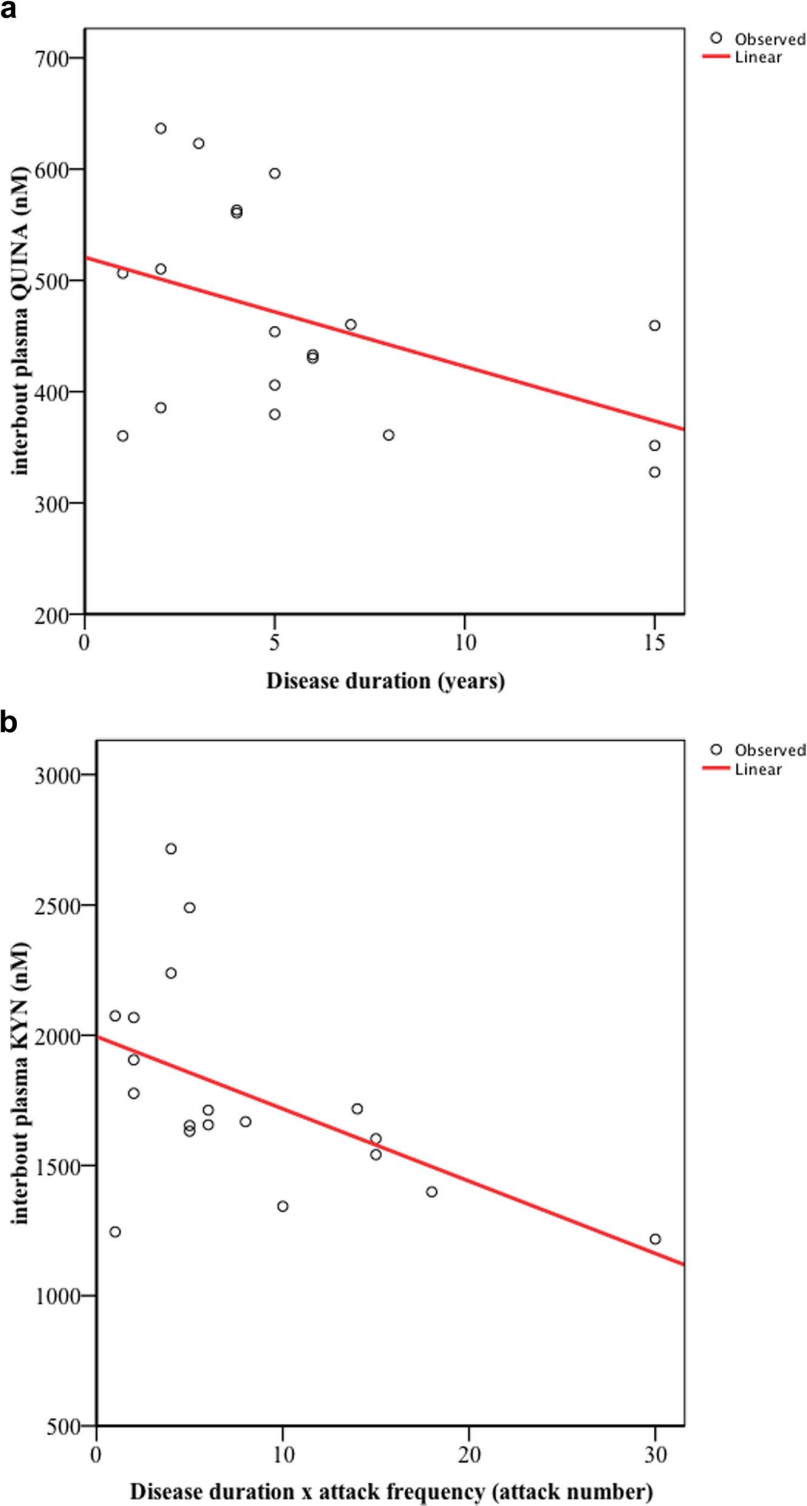
**a** and **b** Significant correlations between the age of healthy controls and plasma levels of Trp, KYNA, 3-HANA and QUINA, as well as the age of CH patients and plasma levels of ictal ANA. Median of data and value of interquartile range were represented in the figures. Trp: tryptophan, KYNA: kynurenic acid, 3-HANA: 3-hydroxyanthranilic acid, QUINA: quinolinic acid, ANA: anthranilic acid

Mild linear relationships were revealed between the disease duration and concentration changes of interbout QUINA levels (*n* = 19; *p* < 0.048, *R* =  − 0.459) **(**Fig. 3a**)**. Moderate association was detected between the total number of CH attacks experienced during the lifetime of patients (disease duration x attack frequency) and the altered interbout KYN concentrations (*n* = 19; *p* < 0.024, *R* =  − 0.516) **(**Fig. 3b**)**. Significant correlations were found using linear regression models between the age of healthy controls and their plasma Trp (*p* < 0.005, *R* =  − 0.682), KYNA (*p* < 0.040, *R* =  − 0.534), 3-HANA (*p* < 0.025, *R* =  − 0.573) and QUINA (*p* < 0.025, *R* =  − 0.574) levels. We found negative association between the ictal concentrations of ANA and the age of CH patients (*n* = 11; (*p* < 0.037, *R* =  − 0.696). The other examined clinical parameters did not show correlation with the plasma concentrations of metabolites.

## Discussion

In this preliminary study, we examined the peripheral Trp metabolism in 24 episodic CH patients and in healthy control subjects, particularly the alterations of plasma kynurenine metabolites between cluster periods, and during headache attack in the cluster phase. We paid special attention to examining whether there are relationships between metabolic changes and the duration of disease, the frequency of attacks, the age, the number of hours spent in a headache, etc. Previously, we identified significantly decreased plasma levels of Trp, KYN, ANA, XA and PICA during the interictal period in episodic migraine patients compared to healthy control subjects. However, little is known about changes of kynurenine metabolites in CH patients.

Our current data show that the plasma level of KYN significantly decreased, while the concentration of QUINA increased during the interbout period of CH patients compared to controls, in contrast to the migraine data, where the entire metabolic route was significantly depressed during the attack-free period. The neuroprotective KYN and the neurotoxic QUINA stand out from the Trp pathway in CH patients.

In order to assess how the observed changes in Trp metabolism would perform in classifying interbout and ictal cluster groups from healthy participants, we conducted a multivariate analysis, PLS-LDA, that uses multiple variables to solve a classification problem. We observed high classification accuracy for all three pairings, which means that Trp metabolites have potential for use in data-driven classification of cluster patients. However, a cardinal limitation in this analysis is the low number of cluster headache patients, which makes out-of-sample validation and the use of more sophisticated machine learning classifier models difficult. Future studies could aim to solve this problem and improve classification by pooling data from multiple headache centers.

The profile of plasma kynurenine metabolites showed declining trend during the interbout period compared to healthy subjects, however there are four exceptions: the 3-HK, the XA and the 3-HANA showed rising tendency, while the level of QUINA was significantly higher during the attack-free phase (Table 3). It means if the concentrations of latter molecules start to rise during the interbout period, it will shift the metabolic balance of Trp pathway toward the toxic direction, subsequently it can initiate attacks, for example they can induce glutamatergic hyperexcitability thorough NMDA-receptors agonism [19, 20]. Meanwhile, the protective function of KYN does not prevail, because its level constantly and significantly decreases during both the attack-free and ictal periods. Since the KYN-QUINA conversion is significant in the interbout period, it will result in neurotoxic compounds are present in increasing quantities when the patients seem to be well, however these processes are the forerunners of the attack. These metabolic changes, especially the accumulation of toxic compounds and other environmental or endogenous factors can be triggers for the appearance of cluster headache [16, 21]. Then the concentration of QUINA returns to the control values, but the level of KYN further decreases. Since we could not measure the alterations of kynurenine metabolites between attacks in the cluster period, it is hypothesized that the decreased KYN concentration is maintained during the cluster period, which can last from weeks to months. However, this bout period is enough long to happens some changes in the lifestyle of patients, which can help to eliminate the attacks. Consequently, the balance of KP can restore at the end of cluster period. There are external and/or internal factors, which can affect the onset and end of headache. 1.) It is known that there are abnormalities in the biological clock of body in CH patients, which is supervised by the hypothalamus and related to the seasonal appearance of disease [22, 23] 2.) Although, the chronic stress, the sleep deprivation, the depression and the hormonal changes, as interrelated agents, are usually not the most relevant provoking factors of CH unlike the migraine, but through the KP can indirectly influence the development and the duration of headache period [24–26] 3.) Certain foods (e.g. Trp-rich diet), drinks (e.g. alcohol) and medications (e.g. nitroglycerine) may also be involved in these processes: the increased Trp intake might be useful to elevate the protective KYN or KYNA levels not only in migraineurs but also in CH patients, but drinking alcohol during the cluster period can increase the risk of severe headache [27, 28]. Therefore the relationship between KP and alcohol consumption confirmed in study of Leclercq and her co-workers can imply theirs association with CH too: increased concentrations of QUINA and decreased levels of KYNA were observed in alcohol use disorder patients. These metabolic alterations are equal with results observed in our CH patients concerning theirs KP [29]. There is a complex relationship between peripheral and central Trp pathways, so it is unknown whether plasma concentrations assessed in our study reflect brain concentrations of metabolites. Speculative theories were summarized in our previous migraine study [15]. If favorable changes can occure during the cluster period, which can re-integrate the balance of KP, it can help to stop the attacks. This is followed by a remission period, when headaches no occurs for months and sometimes even years, then enigmatic trigger causes imbalance in the KP, which results altered glutamate neurotransmisson. These adverse changes can caueses development of attack.

## Summary

Our results provided that the increased QUINA level parallel with the decreased KYN level are triggers of the CH during the interbout period. When the headache attack starts the concentration of KYN further decreases, whilst the level of QUINA returns to the control level. During the cluster period the KYN maintains at low level until the Trp metabolism is resolved as a result of some internal and/or external factors mentioned above. Presumably, the level of KYN returns to the control value at the end of cluster period and later, when starts the following cluster period the concentration of KYN decrease and QUIN increase, which results altered glutamate neurotransmission and hyperexcitability in the central nervous system.

To the exploration of metabolic alterations, it would be necessary to collect plasma samples several times during both the interbout and ictal periods, which contribute to the better understanding of CH pathomechanism.

## Limitations

Limitations of our study: number of samples, particularly the ictal samples, PLS-LDA model requires out-of-sample validation, missing of dietary intake monitoring.

## Supplementary Information

**Additional file 1:** **Supplementary Table1. **Chromatographic data of the measured TRPmetabolites. Operating software was MassLynx V4.2SCN977. 11 calibrators were prepared to this study. Narrower linear range was obtainedin the case of 5-HIAA (first 6 solution). 

**Additional file 2:** **Supplementary Table 2.** Applied MRM transition settings for TRP metabolites utilizing Waters TQ-S Micro MS. Analyte levels in human plasma were determined using data mentioned in this table.

## Data Availability

Data concerning metabolite levels and clinical characteristics are available in the Supplementary file.

## Abbreviations

3-HANA: 3-Hydroxyanthranilic acid
3-HK: 3-Hydroxykynurenine
5-HIAA: 5-Hydroxyindoleacetic acid
5-HT: 5-Hydroxytryptamine
ANA: Anthranilic acid
CH: Cluster headache
Glu: Glutamate
IQR: Interquartile range
KP: Kynurenine pathway
KYN: L-kynurenine
KYNA: Kynurenic acid
MELA: Melatonin
NMDA: N-methyl-D-aspartate
PACAP: Pituitary adenylate cyclase-activating polypeptide
PLS-LDA: Partial least squares-linear discriminant analysis
PICA: Picolinic acid
QC: Quality control
QUINA: Quinolinic acid
Trp: Tryptophan
UHPLC–MS/MS: Ultra high-performance liquid chromatography-tandem mass spectrometry
XA: Xanthurenic acid
