# Supplementary material for: Cluster headache and kynurenines
Source: J Headache Pain. 2023 Apr 5;24(1):35. doi: 10.1186/s10194-023-01570-9 (PMC10074689; doi:10.1186/s10194-023-01570-9)
Supplement: Supplementary file 1 — Additional file 1: Supplementary Table1. Chromatographic data of the measured TRPmetabolites. Operating software was MassLynx V4.2SCN977. 11 calibrators were prepared to this study. Narrower linear range was obtainedin the case of 5-HIAA (first 6 solution). [file 10194_2023_1570_MOESM1_ESM.docx]

**Supplementary Table 1.** Chromatographic data of the measured TRP metabolites. Operating software was MassLynx V4.2 SCN977. 11 calibrators were prepared to this study. Narrower linear range was obtained in the case of 5-HIAA (first 6 solution).

| Metabolite | Retention time (min) | Linear range (ng/mL) | Calibration curve | R^2^ |
| --- | --- | --- | --- | --- |
| 3-HK | 1.40 | 0.625 – 20.0 | 2.53847 x - 0.867225 | 0.9981 |
| KYN | 2.23 | 46.875 – 1 500.0 | 1.81225 x + 4.48078 | 0.9999 |
| 5-HT | 2.28 | 9.375 – 300.0 | 2.43416 x + 5.72209 | 0.9998 |
| 3-HANA | 3.01 | 0.625 – 20.0 | 0.581564 x + 0.00773113 | 0.9990 |
| TRP | 3.39 | 937.5 – 30 000.0 | 17.0744 x + 2285.12 | 0.9998 |
| 5-HIAA | 3.75 | 5.625 – 30.0 | 6.39825 x - 25.2444 | 0.9991 |
| ANA | 3.94 | 0.625 – 20.0 | 1.04401 x + 1.547 | 0.9994 |
| KYNA | 3.97 | 0.938 – 30.0 | 2.33303 x - 0.778719 | 0.9996 |
| QUIN | 4.51 | 4.688 – 180.0 | 0.940585 x + 21.4087 | 0.9994 |
| PICA | 4.69 | 0.625 –20.0 | 1.37843 x + 1.14019 | 0.9977 |
| XA | 4.87 | 0.625 – 20.0 | 2.64667 x + 0.0711093 | 0.9995 |

Chromatographic data of the deuterated internal standards.

| IS | Retention time (min) | Conc. (ng/mL) | SD | IS | Retention time (min) | Conc. (ng/mL) | SD (%) |
| --- | --- | --- | --- | --- | --- | --- | --- |
| d3-3-HK | 1.39 | 20.0 | 4.142 | d5-KYNA | 3.96 | 20.0 | 2.685 |
| d4-KYN | 2.17 | 800.0 | 1.500 | d3-QUIN | 4.51 | 150.0 | 3.129 |
| d4-5-HT | 2.26 | 300.0 | 1.662 | d4-PICA | 4.68 | 20.0 | 14.101 |
| d3-3-HANA | 2.98 | 20.0 | 4.990 | d4-XA | 4.87 | 20.0 | 2.817 |
| d5-TRP | 3.36 | 4 000.0 | 6.055 |  |  |  |  |
| d5-5-HIAA | 3.73 | 40.0 | 5.107 |  |  |  |  |

QC samples – runtime 30.5 hrs (mean concentrations, 15-15 replicates of each, the given n is the samples size of the pooled individual samples).

| Metabolite | Control group (n = 9) | | Cluster headache group (n = 10) | |
| --- | --- | --- | --- | --- |
|  | Concentration (ng/mL) | SD value | Concentration (ng/mL) | SD value |
| 3-HK | 8.254 | 0.487 | 9.960 | 0,526 |
| KYN | 353.420 | 2.826 | 387.036 | 3.555 |
| 5-HT | 4.087 | 0.908 | 36.497 | 0.815 |
| 3-HANA | 7.241 | 0.400 | 9.504 | 0.544 |
| TRP | 12 793.123 | 527.794 | 12 558.435 | 544.574 |
| 5-HIAA | 8.376 | 0.917 | 8.991 | 0.755 |
| ANA | 5.627 | 0.470 | 4.209 | 0.277 |
| KYNA | 6.615 | 0.170 | 6.709 | 0.160 |
| QUIN | 52.144 | 2.239 | 54.888 | 2.640 |
| PICA | 4.319 | 0.736 | 3.371 | 0.857 |
| XA | 2.865 | 0.158 | 2.750 | 0.144 |

PICA, QUIN, and XA were analysed by their mono butylated forms.

According to the absence of relative internal standard for ANA, d5-KYA was applied to the quantification process in each sample, as their retention times are comparable.

Timing of the QC samples

5 replicates of control QC ⭢ 5 replicates of cluster QC ⭢ duplicates of the standards ⭢ 1-1 QC of both groups ⭢ (duplicates of 7 or 8 randomized samples ⭢ 1-1 QC) x 4 ⭢ duplicates of vortexed, same standards ⭢ 1-1 QC of both groups ⭢ (duplicates of 7 or 8 randomized samples ⭢ 1-1 vortexed, same QC) x 4

QC samples – range of measured concentration levels.

| Compound | Control group (n = 9) | | Cluster headache group (n = 10) | |
| --- | --- | --- | --- | --- |
|  | Min. value (ng/mL) | Max. value (ng/mL) | Min. value (ng/mL) | Max. value (ng/mL) |
| 3-HK | 7.554 | 9.078 | 9.044 | 10.912 |
| KYN | 350.225 | 359.489 | 382.282 | 396.047 |
| 5-HT | 2.306 | 5.766 | 34.890 | 37.935 |
| 3-HANA | 6.599 | 7.858 | 8.055 | 10.238 |
| TRP | 11 849.924 | 13 663.727 | 11 734.006 | 13 261.491 |
| 5-HIAA | 6.942 | 9.755 | 7.778 | 10.342 |
| ANA | 4.809 | 6.622 | 3.698 | 4.624 |
| KYNA | 6.336 | 6.879 | 6.450 | 6.951 |
| QUIN | 48.922 | 56.465 | 50.904 | 60.022 |
| PICA | 3.048 | 5.687 | 2.146 | 4.910 |
| XA | 2.465 | 3.061 | 2.564 | 2.988 |
