# Supplementary material for: Cluster headache and kynurenines
Source: J Headache Pain. 2023 Apr 5;24(1):35. doi: 10.1186/s10194-023-01570-9 (PMC10074689; doi:10.1186/s10194-023-01570-9)
Supplement: Supplementary file 2 — Additional file 2: Supplementary Table 2. Applied MRM transition settings for TRP metabolites utilizing Waters TQ-S Micro MS. Analyte levels in human plasma were determined using data mentioned in this table. [file 10194_2023_1570_MOESM2_ESM.docx]

**Supplementary Table 2.** Applied MRM transition settings for TRP metabolites utilizing Waters TQ-S Micro MS. Analyte levels in human plasma were determined using data mentioned in this table.

| Analyte | Precursor ion [M+H]^+^ | Cone voltage (V) | Quantifier ion [M+H]^+^ | CE_1_ (eV) | Qualifier ion [M+H]^+^ | CE_2_ (eV) | Retention window (min) |
| --- | --- | --- | --- | --- | --- | --- | --- |
| 3-HK | 225.05 | 2 | 207.94 | 8 | 161.96 | 18 | 1.1 – 1.8 |
| d3-3-HK | 228.07 | 6 | 210.90 | 8 | 111.10 | 12 |  |
| KYN | 209.05 | 6 | 191.94 | 8 | 93.93 | 12 | 1.9 – 2.6 |
| d4-KYN | 213.08 | 2 | 196.02 | 8 | 97.97 | 14 |  |
| 5-HT | 177.06 | 24 | 160.01 | 10 | 132.04 | 20 | 2.0 – 2.7 |
| d4-5-HT | 181.03 | 14 | 164.04 | 8 | 136.03 | 22 |  |
| 3-HANA | 154.01 | 16 | 107.93 | 18 | 79.95 | 26 | 2.7 – 3.5 |
| d3-3-HANA | 156.97 | 4 | 138.96 | 12 | 82.94 | 28 |  |
| TRP | 205.06 | 12 | 188.01 | 8 | 145.92 | 16 | 3.2 – 3.8 |
| d5-TRP | 210.10 | 8 | 150.06 | 18 | 122.03 | 26 |  |
| 5-HIAA | 192.03 | 23 | 145.97 | 12 | 91.00 | 34 | 3.5 – 4.1 |
| d5-5-HIAA | 197.07 | 2 | 150.18 | 12 | 122.06 | 30 |  |
| ANA | 137.95 | 14 | 119.90 | 8 | 91.93 | 22 | 3.8 – 4.5 |
| KYNA | 190.01 | 6 | 143.95 | 16 | 115.98 | 30 | 3.7 – 4.5 |
| d5-KYNA | 195.05 | 2 | 148.98 | 18 | 121.00 | 32 |  |
| QUIN_e_ | 224.05 | 2 | 77.93 | 30 | 149.95 | 12 | 4.3 – 4.9 |
| d3-QUIN_e_ | 227.08 | 2 | 153.02 | 12 | 80.99 | 30 |  |
| PICA_e_ | 180.06 | 2 | 105.91 | 14 | 123.94 | 10 | 4.5 – 5.1 |
| d4-PICA_e_ | 184.09 | 2 | 127.97 | 10 | 109.88 | 14 |  |
| XA_e_ | 262.07 | 2 | 159.99 | 26 | 205.99 | 14 | 4.7 – 5.3 |
| d4-XA_e_ | 266.10 | 2 | 164.02 | 26 | 210.02 | 14 |  |

These settings were also included in the Experimental Setup, but were not utilized in the qualification method:

| Analyte | Precursor ion [M+H]^+^ | Cone voltage (V) | Quantifier ion [M+H]^+^ | CE_1_ (eV) | Qualifier ion [M+H]^+^ | CE_2_ (eV) | Retention window (min) |
| --- | --- | --- | --- | --- | --- | --- | --- |
| 3-HK_e_ | 281.11 | 2 | 151.98 | 14 | 109.88 | 26 | 4.1 – 4.7 |
| d3-3-HK_e_ | 284.13 | 2 | 155.03 | 14 | 112.02 | 26 |  |
| QUIN_2e_ | 280.12 | 2 | 77.93 | 36 | 105.91 | 26 | 4.8 – 5.4 |
| d3-QUIN_2e_ | 283.14 | 2 | 108.96 | 26 | 80.98 | 36 |  |

Additionally, a full MS scan was scheduled with the above detailed retention windows from 1 to 6 minutes in the range of 50-400 m/z at a cone voltage of 20V with a 0.1 scan/sec speed.

Every setup was based on IntelliStart generated results and was optimized/tested with injections as well.

Meaning of lowering indices: e = mono butyl ester form, 2e = di butyl ester form, CE_1_ = Collision energy of Quantifier ion, CE_2_ = Collision energy of Qualifier (or Target) ion.
